# Evaluation of the Body Burden of Short- and Medium-Chain Chlorinated Paraffins in the Blood Serum of Residents of the Czech Republic

**DOI:** 10.3390/jox14040107

**Published:** 2024-12-18

**Authors:** Denisa Parizkova, Aneta Sykorova, Jakub Tomasko, Ondrej Parizek, Jana Pulkrabova

**Affiliations:** Department of Food Analysis and Nutrition, Faculty of Food and Biochemical Technology, University of Chemistry and Technology Prague, Technicka 3, 166 28 Prague, Czech Republic; denisa.turnerova@vscht.cz (D.P.); sykorovc@vscht.cz (A.S.); jakub.tomasko@vscht.cz (J.T.); parizekn@vscht.cz (O.P.)

**Keywords:** chlorinated paraffins, human biomonitoring, endocrine disruptor

## Abstract

Short- and medium-chain chlorinated paraffins (SCCPs and MCCPs) are environmental contaminants known for their persistence and bioaccumulation in fatty tissues. SCCPs are considered potential carcinogens and endocrine disruptors, with similar effects expected for MCCPs. This study investigated the body burden of SCCPs and MCCPs in residents of two regions of the Czech Republic with different levels of industrial pollution. Blood serum samples from 62 individuals in Ceske Budejovice (control area) and Ostrava (industrial area) were analysed. The results showed higher concentrations of SCCPs (<120–650 ng/g lipid weight (lw)) and MCCPs (<240–1530 ng/g lw) in Ostrava compared to Ceske Budejovice (SCCPs: <120–210 ng/g lw, MCCPs: <240–340 ng/g lw). The statistical analysis revealed no significant correlations between chemical concentrations and demographic variables such as age, BMI, or gender. The findings are consistent with European and Australian studies but significantly lower than levels reported in China. This is the first comprehensive survey of SCCPs and MCCPs in human blood serum in the Czech Republic and the second study in Europe. The data collected in this study are essential for assessing SCCPs and MCCPs. They will contribute to a better understanding the potential health risks associated with exposure to these chemicals.

## 1. Introduction

Man-made chemical pollution is one of the current civilisation problems. Chemicals enter the environment, food, and living organisms during production, use, and disposal, contributing to human exposure [1].

One of the groups of environmental pollutants currently under discussion are chlorinated paraffins (CPs). CPs are complex synthetic mixtures of polychlorinated n-alkanes with different carbon chain lengths and chlorine contents in the molecules. Commercially produced technical CPs are usually classified into three groups according to the length of the carbon chain: short-chain chlorinated paraffins (SCCPs, C10–C13), medium-chain chlorinated paraffins (MCCPs, C14–C17) and long-chain chlorinated paraffins (LCCPs, C18–C30). The molecule’s chlorination degree is usually 30–70 weight percentages [2]. However, the range of industrial products associated with CPs can be constantly expanded. Recently, CPs with shorter chains than SCCPs have also been discovered, so-called very short-chain CPs (vSCCPs, C6–C9), which occur as impurities in synthetic CP products or by-products [3].

Technical mixtures are used as secondary plasticisers and flame retardants in polyvinyl chloride (PVC) products and other plastics, rubber, paints, sealants, and adhesives. These mixtures have also been added to cutting fluids as lubricants and additives that withstand high pressures. They have also been used to dissolve grease in the working and processing of leather for clothing, fabrics and furniture [4]. SCCPs have been classified as potential human carcinogens [5], and due to their persistence in the environment, ability to bioaccumulate, and possible adverse effects, they were classified as persistent organic pollutants (POPs) listed in the Stockholm Convention in 2017 [6]. MCCPs show similar properties and have been proposed as candidate compounds for inclusion in the Convention since 2021. LCCPs are also thought to have these properties, but little is known about them. Studies on vSCCPs associated with commercial products and environmental and human blood samples can already be found in the literature [3].

Since SCCPs and MCCPs are used as plasticisers in plastics, sources of exposure may include these plastics used in products that come into contact with food (hand blenders, plastic bottles, and food packaging) or toys and their packaging [7,8]. They are also used in insulation materials, which may pose a risk during manufacture or disposal. They have also been found in textiles such as T-shirts and socks, which may be a source of dermal exposure [9].

CPs have been detected in all environmental compartments—water [10,11,12], soil [13], sediments [14,15,16], air [17,18,19], dust [20], food and crops [21,22,23,24,25]. The most important exposure source is dietary intake, i.e., ingestion of contaminated food or food supplements [26,27], followed by inhalation exposure via indoor air and dermal exposure from dust and consumer products [28]. Breast milk is the most crucial way of infants’ exposure to CPs, which reflects the mother’s internal exposure [29].

Human biomonitoring is a method that focuses on determining internal exposure to CPs and other pollutants. This method uses human biological samples to measure xenobiotics and their metabolites. The preferred and common sample type for assessing internal exposure to CPs is human blood plasma or serum, as blood is in contact with all organs and tissues [30]. Breast milk is also used to assess the exposure of both the infant and the mother [31]. However, hair and nails can also be used to evaluate exposure [30]. Currently, three European studies on the CPs in human serum are available. One of them is from the Czech Republic, where the medians were 370 ng/g lipid weight (lw) and 360 ng/g lw (SCCPs and MCCPs) [32]. Unlike the first study, samples were taken from both genders, and it was possible to consider this parameter when processing the results. The other two are from Norway. In one of these studies, the medians were 2500 ng/g lw for SCCPs and 1100 ng/g lw for MCCPs [33]. Data are also available for the Australian population. The reported median concentrations were 97 ng/g lw and 190 ng/g lw (SCCPs and MCCPs, respectively) [34]. Concentrations of SCCPs and MCCPs in blood plasma, serum, and breast milk have been reported mainly from China. There, mean values for blood serum range from 13,800 to 20,949 ng/g lw for SCCPs and from 1340 to 15,200 ng/g lw for MCCPs [35,36,37]. Results from Europe and Australia are up to 50-times lower than in China, which is consistent with China being the largest producer of CPs in the world [2]. The study aimed to determine human blood serum samples (n = 62) from men and women for the presence of SCCPs and MCCPs, thereby extending the results from Europe, which are still few.

## 2. Materials and Methods

In this survey, we aimed to further confirm the results of a pilot methodological study [32] by analysing a broader set of samples. The studied general population is from two locations (the industrial area of Ostrava and the control area of Ceske Budejovice) in the Czech Republic. Gas chromatography coupled with high-resolution mass spectrometry operated in negative chemical ionisation mode (GC-NCI-HRMS) was used to determine CPs in human blood serum samples (n = 62). In the pilot study, samples were collected only from men, but in this survey, we had blood serum sampled from both men and women.

### 2.1. Analytical Standards and Chemicals

Single-chain standard mixtures of SCCPs with 45%, 55%, and 65% chlorine (*w*/*w*) and single-chain mixtures of MCCPs with 45% and 55% chlorine (*w*/*w*) (in 10 and 100 µg/mL cyclohexane, respectively) were purchased from LGC Standards (Teddington, UK). Polychlorinated biphenyl 166 (10 µg/mL isooctane), used as an internal standard, was purchased from Dr. Ehrenstorfer (Augsburg, Germany). Other chemicals used are listed in the earlier study by Tomasko et al., 2021. The polypropylene test tubes (50 mL) used for the extraction were obtained from Eppendorf (Hamburg, Germany).

### 2.2. Samples

The current study determined SCCPs and MCCPs in human blood serum samples collected from the Czech adult population (n = 62) between September 2019 and May 2021. The cohort was a healthy population with no significant health problems. The samples were obtained as a part of the HAIE (Healthy Aging in Industrial Environment) project, which was conducted in close collaboration with the Institute of Experimental Medicine of the Czech Academy of Sciences. The HAIE project aimed to determine the links between industrial pollution and human health. The HAIE project was not primarily focused on CPs, but beyond the project’s scope, this unique sample set was used to assess the exposure of the Czech population to CPs. Serum samples were collected from two locations—Ostrava (49°50′ N 18°17′ E) and Ceske Budejovice (48°58′ N 14°28′ E). Ostrava was selected as a typical industrial area where the population is likely to be more exposed to CPs than Ceske Budejovice, which was chosen as a control area, similar to previous studies focusing on the presence of persistent organic pollutants (POPs) in human biological samples in the Czech Republic [38,39]. Donors of volunteer samples were men (n = 34) and women (n = 28) aged 18–63. The detailed information of individual volunteers includes age, BMI, gender, occupation, and education, which are summarised in the Supplementary Materials in Table S1.

### 2.3. Sample Preparation Procedure

For sample preparation, the analytical approach from the study by Svarcova et al., 2019 [40] was used, which was initially developed for a broad group of halogenated pollutants (polychlorinated biphenyls, organochlorine pesticides, halogenated flame retardants) with minor modifications for CPs described in the study by Tomasko et al., 2021 [32]. Three grams of blood serum was mixed with 0.04 mL of internal standard stock solution in acetone (PCB 166 at 50 ng/mL used for CPs analysis). The procedure involved a triplicate extraction with a mixture of 6 mL n-hexane:diethyl ether (9:1, *v*/*v*). Anhydrous sodium sulfate was used to remove residual water. Solid phase extraction (SPE) on a Florisil^®^ column (0.5 g) was applied as a clean-up step. The sample was dissolved in 0.25 mL isooctane. The total lipid content was determined enzymatically.

### 2.4. Instrumental Analysis and Quantification of SCCPs and MCCPs

Instrumental analysis was performed by gas chromatography coupled with high-resolution mass spectrometry (GC-HRMS) using an Agilent 7200B system consisting of Agilent 7890B gas chromatograph equipped with a multimode inlet, PAL RSI 85 autosampler for automated injection, and quadrupole-time of flight mass spectrometer (GC/Q-TOF; Agilent Technologies, Santa Clara, CA, USA) in negative chemical ionisation mode. The instrument parameters and quantification algorithm are documented in Tomasko et al., 2023 [9].

### 2.5. QA/QC

A previously validated and established method was used to determine SCCPs and MCCPs in human blood serum. The validation of the procedure for the analysis of SCCPs and MCCPs in human blood serum is described in a previous study by Tomasko et al., 2021 [32] (recoveries in the range of 71–89%, repeatability < 20%—expressed as relative standard deviation, RSD). The LOQ was set to the value in standards where at least three congener groups were detected (120 ng/g lw and 240 ng/g lw for SCCPs and MCCPs, respectively). The value LOD is then taken as one-third of the value LOQ.

Procedural blanks were prepared each day. The SCCPs and MCCPs concentrations in the blanks ranged from LOD to LOQ; the blank CPs values were subtracted from the samples. The polypropylene tubes used in the extraction process have been previously identified [32] as a critical point in background contamination of the laboratory with CPs. In this study, every batch of tubes was tested and did not contain detectable CPs.

To monitor the performance characteristics of the method on each day of extraction, blank samples of bovine serum were spiked with CPs to a concentration of 50 ng/g weight and 100 ng/g weight for SCCPs and MCCPs, respectively. The mean recoveries were 116% and 122% (SCCPs and MCCPs, respectively). We can not analyse certified reference material because it is not available. The only available reference material is fish tissue.

### 2.6. Statistical Analyses

MetaboAnalyst 5.0 (University of Alberta, Edmonton, AB, Canada) and RStudio (Posit, Vienna, Austria) software were used for statistical data processing. Statistically significant results were set as α = 0.5. Data were log-transformed as they did not have a normal distribution, which was tested using the Shapiro-Wilk normality test at the α = 0.05 level. Welch’s Two Sample *t*-tests were used to assess differences in the levels of contaminants between sampling sites. Spearman correlation tests were used to identify possible significant correlations in the measured data set. One-way and two-way variance analyses (ANOVA) were used to compare the distribution of age groups, BMI, and CP.

For statistical analysis of the results in human blood sera, concentrations below the LOQ (120 ng/g lw for SCCPs and 240 ng/g lw for MCCPs) were replaced by half the value of LOQ (60 ng/g lw for SCCPs and 120 ng/g lw for MCCPs) and values below the LOD (40 ng/g lw for SCCPs and 80 ng/g lw for MCCPs) were replaced by half the value of LOD (20 ng/g lw for SCCPs and 40 ng/g lw for MCCPs). The half values are used to identify possible differences between the groups, as it is often not possible to assess population exposure based on the different groups due to the low concentrations. This procedure was used for all statistical calculations.

## 3. Results

### 3.1. Frequency of Detection of CPs in Human Blood Samples

**Figure 1:**
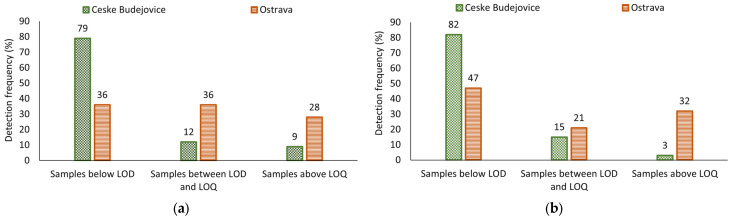
The frequency of findings above the LOQ/LOD values (**a**) The frequency of findings above the LOQ/LOD of SCCPs in control area (Ceske Budejovice) and industrial area (Ostrava); (**b**) The frequency of findings above the LOQ/LOD of MCCPs in control area (Ceske Budejovice) and industrial area (Ostrava).

The frequency of findings above the LOQ/LOD values (values above the LOQ) of SCCPs was three times lower in Ceske Budejovice (9%) than in Ostrava (28%) as shown in Figure 1a and for MCCPs ten times lower in Ceske Budejovice (3%) than in Ostrava (32%), as shown in Figure 1b. These results could indicate a higher population exposure in Ostrava.

### 3.2. SCCPs and MCCPs Concentrations in Blood Samples from the Czech Republic

**Figure 2:**
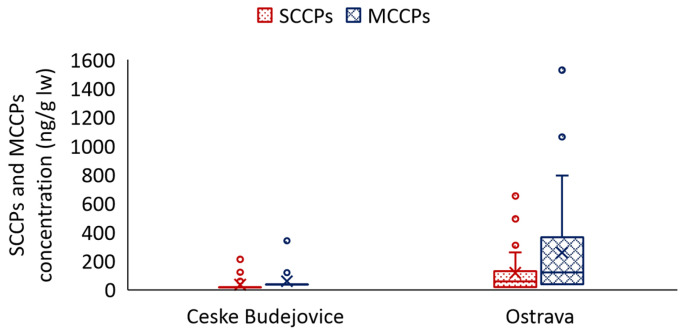
Concentrations of SCCPs and MCCPs in blood serum in both localities. The boxplots illustrate the lower quartile (25% of the results, Q1) or the value that defines the lowest quarter of the values, and the upper quartile (75% of the results, Q3) or the value that separates the highest quarter. The so-called “whiskers” indicate the minimum and maximum values that fall within a range that results from multiplying the value by 1.5 and the variance between Q3 and Q1. The values outside the boxplot with whiskers are outliers (circle). The line between Q1 and Q3 indicates the median, while the cross in this case indicates the mean value.

A statistically significant difference (*p* < 0.05) was found for the individual locations in the Czech Republic (Figure 2). In Ceske Budejovice, measured concentrations ranged from <120–210 ng/g lw for SCCPs (3 samples above LOQ) and <240–340 ng/g lw for MCCPs (1 sample above LOQ), and in Ostrava <120–650 ng/g lw for SCCPs (8 samples above LOQ) and <240–1530 ng/g lw for MCCPs (9 samples above LOQ).

### 3.3. Correlation Between SCCPs and MCCPs Concentrations in Serum and Gender, Age and BMI

When looking for a correlation between the concentrations of SCCPs and MCCPs and the gender of the Czech participants, no statistically significant difference was found. Since CPs are persistent and can accumulate in human adipose tissue, a correlation between the measured concentrations and the age of the study participants was searched for. In this case, there was no statistically significant difference between the concentrations of SCCPs and MCCPs and the age of the donors. Consistent with the assumption of an accumulation of CPs in adipose tissue, the relationship between SCCPs and MCCPs concentrations and the BMI of the study participants was examined. This test also showed no statistically significant difference.

The *p*-values and correlation coefficients are shown in Table 1.

**Figure 3:**
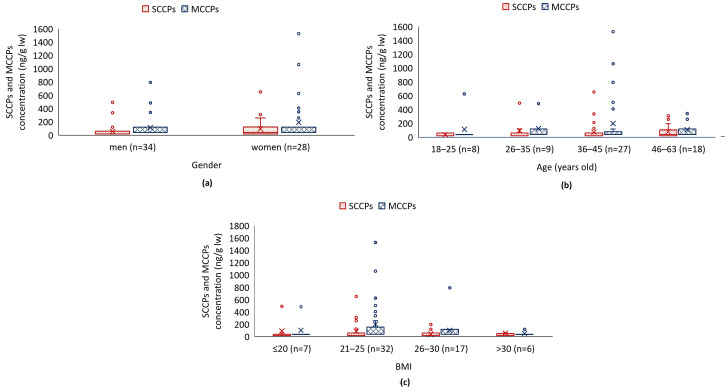
Concentration of SCCPs and MCCPs in blood serum by gender (**a**), concentration of SCCPs and MCCPs in blood serum in age groups (**b**) and concentration of SCCPs and MCCPs in blood serum in BMI groups (**c**). The boxplots illustrate the lower quartile (25% of the results, Q1) or the value that defines the lowest quarter of the values, and the upper quartile (75% of the results, Q3) or the value that separates the highest quarter. The so-called “whiskers” indicate the minimum and maximum values that fall within a range that results from multiplying the value by 1.5 and the variance between Q3 and Q1. The values outside the boxplot with whiskers are outliers (circle). The line between Q1 and Q3 indicates the median, while the cross in this case indicates the mean value.

Figure 3a illustrates the measured concentrations of SCCPs and MCCPs for both male and female participants. The measured concentrations of SCCPs and MCCPs in men’s serum ranged from 120 to 494 ng/g lw (three samples above the LOQ) and from 240 to 794 ng/g lw (four samples above the LOQ), respectively. For women, the measured concentrations ranged from <120–650 ng/g lw for SCCPs (eight samples above LOQ) and <240–1530 ng/g lw for MCCPs (six samples above LOQ). The median concentrations in both groups were below the LOQ for SCCPs and MCCPs.

As a function of age, the concentrations of SCCPs and MCCPs are presented in Figure 3b. The highest concentration of both compounds was observed in the 36–45 age group. The median concentrations of SCCPs and MCCPs in all groups are below the limit of quantification (LOQ).

The results of the measurement of SCCPs and MCCPs concentrations, divided based on BMI, are presented in Figure 3c. The highest concentration of SCCPs and MCCPs was observed in the 21–25–45 BMI group. The median concentrations of SCCPs and MCCPs in all groups were below the limit of quantification (LOQ).

The absence of a trend between CP concentrations and age or BMI may be related to several factors, including excretion rate, metabolism, environmental exposure, and the composition of chlorinated paraffins.

In the study participants, some occupations could potentially be more exposed, such as firefighter, Plastics specialist, and Foundry worker in an ironworks. Unfortunately, we do not have a detailed job description of the individual employees. Higher concentrations were confirmed for individuals with the employment of foundry worker in an ironworks (31 years old, man, high concentrations of both SCCPs (494 ng/g lw) and MCCPs (489 ng/g lw)), but as mentioned, it is not possible to identify employment as a source of exposure. For example, for firefighters, the concentrations of SCCP and MCCP were below the LOQ, and the same finding was found for the plastics specialist. The most exposed participants were a policewoman (42 years old, woman, high concentration of both SCCPs (653 ng/g lw) and MCCPs (1064 ng/g lw)), a teacher (38 years old, woman, high concentration of MCCPs (1529 ng/g lw)) and a foundry worker in an ironworks. Occupational information for all study participants is provided in Table S1.

### 3.4. SCCPs and MCCPs Homologue Profiles in Serum Samples

**Figure 4:**
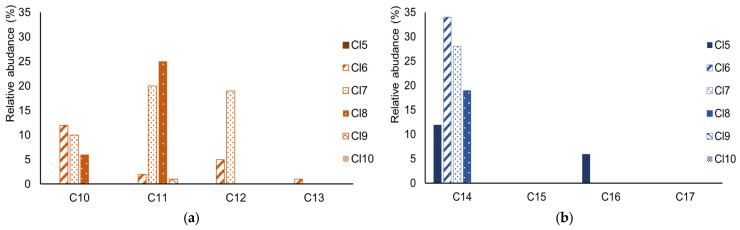
Relative abundance of CPs (**a**) Relative abundance of SCCPs congener groups in blood serum samples from the Czech Republic; (**b**) Relative abundance of MCCPs congener groups in blood serum samples from the Czech Republic.

The group of congeners with 11 carbons was the most abundant (48%) in the serum samples, followed by 10 carbons (28%), 12 carbons (23%), and the group of congeners with 13 carbons (1%) was the least frequently detected (Figure 4a). The concentration range (of all samples) of C10, C11 was <LOD—310 ng/g lw, <LOD—288 ng/g lw and <120 ng/g lw for C12 and C13. The median values (of all samples) of C10, C11, C12 and C13 were below LOD.

In the case of MCCPs, the group of congeners with 14 carbons was the most abundant (96%), followed by those with 16 carbons (4%). The groups of congeners with 15 and 17 carbons were not detected at all (Figure 4b). A homologue profile for SCCPs and MCCPs was generated from all samples that showed positive signals for at least two chains. In this study, we already had single-chain standards for MCCP available, so it was possible to extend the pilot study with these results. The concentration range for C14 was from LOD to 1063 ng/g lw; for C16, it was from LOD to 500 ng/g lw, and for C15 and C17 was <240 ng/g lw. The median values (of all samples) of C14, C15, C16 and C17, were all below LOD. The medians of all chains for both Ceske Budejovice and Ostrava were <LOD.

## 4. Discussion

**Figure 5:**
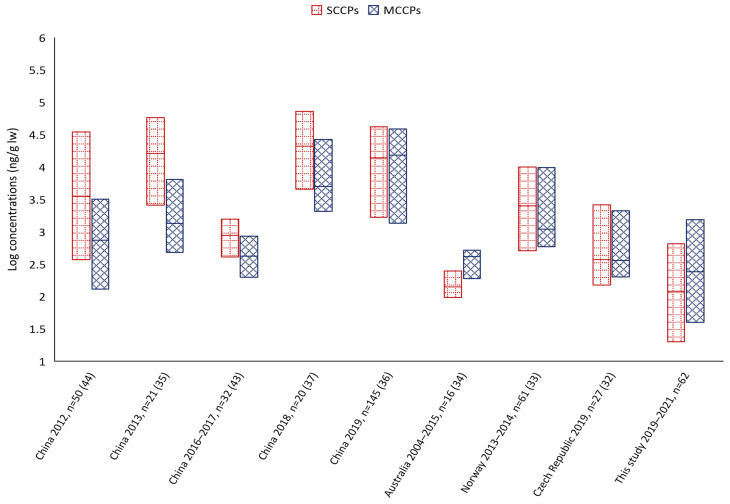
Comparison of SCCP and MCCP concentrations in blood serum based on published studies.

Based on the measured SCCP and MCCP levels at two different sites in the Czech Republic, it became clear that areas with higher industrial activity had higher CP levels, confirming the likely link between exposure to these pollutants and industrial emissions. In the surroundings of Ceske Budejovice, where industrial activity is significantly lower, exposure to CPs and other persistent organic pollutants (POPs) is significantly lower than in the more industrialised areas of the Czech Republic. Ostrava and Karvina are considered the most industrialised regions in the country due to the historical heritage of heavy industry, the current metallurgy and engineering sector and the significant influence of transport [38,39]. While slightly elevated concentrations of SCCPs were found in the blood sera of women, a larger sample with an equal representation of women and men would be required to establish a statistically significant association. In general, it remains difficult to detect statistically significant differences between SCCPs and MCCPs concentrations and the variables gender, age and BMI in the blood serum donors, similar to the studies by Xu et al., 2019 [29], Ding et al., 2020 [36] and Niu et al., 2023 [41]. Compared to the blood serum concentrations reported in peer-reviewed studies, especially from China, the results from samples in the Czech Republic are significantly lower. In China, the concentrations are two orders of magnitude higher, suggesting that the Czech population is exposed to considerably lower amounts than the Chinese population. Samples from Australia [34], collected over a more extended period, showed median concentrations similar to those reported from the Czech Republic. From Europe, only two studies from Norway [33,42] are available, the results of which also indicate a higher exposure of the Norwegian population compared to that in the Czech Republic. In summary, the available studies suggest that people in the Czech Republic are significantly less exposed to CPs than in other countries. Figure 5 shows trends over time, particularly the increase in MCCP concentrations observed in both the Chinese population and the global results.

The figure shows the minimum, median and maximum concentrations. The figure also documents when the samples were taken and how many were provided for the study.

When comparing CP profiles from different studies, it becomes clear that there are fluctuations in the dominant congeners of SCCPs. In some studies, the C10 congener is the predominant form, whereas in others, the C13 congener is the more prevalent. This variability indicates that the sources of SCCPs exposure may be different. In contrast, the C14 congener predominates in MCCPs in several studies [32,35,36,37,42,43,44].

(Chen et al., 2022) [45] reported that in Europe and the USA, the C11 and C12 congeners dominate in technical mixtures and are mainly used in metalworking fluids. In China, conversely, the C10 congener was most prevalent in technical mixtures, primarily employed as additives in PVC products. This indicates that the origin of SCCPs is primarily to be found in industry and only secondarily in products containing SCCPs. Among the MCCPs, the most widely produced and used congener is C14, with China and the USA leading in production. In addition, C15 and C16 congeners are made in China and Europe. When analysing the MCCPs congener groups, it becomes clear that products containing MCCPs originate predominantly from China.

In general, CPs in metalworking fluids have a higher potential for environmental emissions than CPs in PVC products, which have a lower potential for environmental emissions. PVC products release CPs into the environment on a long-term and continuous basis.

There are minimal or almost no restrictions to limit the production and use of CPs in China. As a major producer, China contributes significantly to CPs pollution and environmental emissions. CPs can spread through the air, travel long distances, and eventually enter water bodies that are carried by ocean currents, mainly affecting Japan and South Korea. CPs can also be emitted by products imported from China.

It is worth noting that a transition from SCCPs to MCCPs or even LCCPs is evident in the data from various studies. However, despite the increasing production and use of CPs and the associated increase in environmental exposure, there is still insufficient data to comprehensively assess the exposure of the general population and specific population subgroups. Although more and more scientific publications deal with CPs-related topics, reference values (RV95) for estimating exposure in different population groups are still difficult to find. Future research on CPs must prioritise acquiring further data on their occurrence in the environment, food, everyday objects, and biomonitoring. These comprehensive data sets must be made available to assess the exposure of different population groups and address the ongoing challenges posed by CPs.

## 5. Conclusions

The present study, conducted in the Czech Republic, aimed to analyse the presence of CPs in human blood serum samples from two locations: Ceske Budejovice and Ostrava. The results showed a statistically significant difference in CP concentrations between these areas, suggesting that CPs exposure is likely related to industrial activities, especially in Ostrava, a region with a long history of heavy industry.

Concentrations of SCCPs and MCCPs in serum samples from Ceske Budejovice were generally lower, with SCCPs ranging from <120–210 ng/g lw (9% of samples above LOQ) and MCCPs < 240–340 ng/g lw (3% of samples above LOQ). In contrast, Ostrava had higher CP levels, with SCCPs in the range of <120–650 ng/g lw (29% of samples above LOQ) and MCCPs < 240–1530 ng/g lw (32% of samples above LOQ).

Despite analysing various demographic factors such as gender, age and BMI, the study did not find a clear correlation between these factors and CP concentrations in serum samples.

Although this is the second study on SCCPs and MCCPs in human blood serum from the Czech Republic, this is the first study in which we were able to investigate the presence of SCCPs and MCCPs in human blood serum from women. The results of this study are consistent with the pilot study’s results and confirm the data’s consistency. By increasing the number of samples and including a broader population, this study contributes to understanding SCCPs and MCCPs in human blood serum in Europe and provides important information for risk assessment. The concentrations found in the Czech Republic are significantly lower than in similar studies in China, emphasising the regional differences in exposure to CPs. The results from Australia are more in line with our study than the Norwegian studies, where the results were slightly higher. This study emphasises the importance of monitoring and assessing the presence of CPs in human serum, especially in areas with industrial activities, to better understand potential health risks associated with CPs exposure.

## Figures and Tables

**Table 1:** Values of statistical analysis to look for possible correlations between SCCPs and MCCPs and various factor.

	*p*-Value		Correlation Coefficient	
Search Factor	SCCPs	MCCPs	SCCPs	MCCPs
Gender	0.1027	0.4699	−0.2092	−0.0935
Age	0.3630	0.5499	0.1175	0.0774
BMI	0.9418	0.3001	0.0095	−0.1337

## Data Availability

Data are available by reasonable request to the author.

## Supplementary Materials

The following supporting information can be downloaded at: https://www.mdpi.com/article/10.3390/jox14040107/s1, Table S1: Data about participants of the study.
