# Supplementary material for: Evaluation of the Body Burden of Short- and Medium-Chain Chlorinated Paraffins in the Blood Serum of Residents of the Czech Republic
Source: J Xenobiot. 2024 Dec 18;14(4):2003–14. doi: 10.3390/jox14040107 (PMC11676474; doi:10.3390/jox14040107)
Supplement: Supplementary file 1 [file jox-14-00107-s001.zip › jox-3286849-supplementary.pdf]

Supplementary Materials for

Evaluation of the Body Burden of Short- and Medium-Chain Chlorinated Paraffins in the Blood Serum of Residents of the Czech Republic

Denisa Parizkova, e-mail: denisa.turnerova@vscht.cz

Aneta Sykorova, e-mail: sykorovc@vscht.cz

Jakub Tomasko, e-mail: jakub.tomasko@vscht.cz

Ondrej Parizek, e-mail: parizekn@vscht.cz

Jana Pulkrabova *, e-mail: jana.pulkrabova@vscht.cz

University of Chemistry and Technology, Prague, Faculty of Food and Biochemical Technology, Department of Food Analysis and Nutrition, Technicka 3, 166 28 Prague 6, Czech Republic

* Corresponding author Tel.: +420 220 443 272

Table S1: Data about participants of the study

Sample number	Location	Gender	Age (years)	BMI	Profession	SCCPs (ng/g lw)	MCCPs (ng/g lw)	Education
1	Ceske Budejovice	man	41	20.2	Firefighter	<LOD	<LOD	University
2	Ceske Budejovice	man	43	23.1	Head of Logistics	<LOD	<LOD	High school
3	Ceske Budejovice	man	41	28.7	Financial advisor	<LOD	<LOD	University
4	Ceske Budejovice	man	37	27.1	Soldier	<LOD	<LOD	High school
5	Ceske Budejovice	man	39	24.4	Paramedic	<LOD	<LOD	High school
6	Ceske Budejovice	man	36	21.6	Plastics specialist	<LOD	<LOD	University
7	Ceske Budejovice	man	45	29.2	Officer	<LOD	<LOD	High school
8	Ceske Budejovice	man	53	22.7	Expediter	<LOD	<LOQ	High school
9	Ceske Budejovice	woman	40	19.9	IT consultant	<LOD	<LOD	High school
10	Ceske Budejovice	woman	47	21.2	Director	<LOD	<LOQ	University
11	Ceske Budejovice	man	36	27.7	Military rescuer	<LOD	<LOD	University
12	Ceske Budejovice	woman	28	33.5	Teacher	<LOD	<LOD	University
13	Ceske Budejovice	woman	42	23.1	Production master	<LOD	<LOD	University
14	Ceske Budejovice	man	63	29.3	Methodologist	<LOD	<LOQ	University
15	Ceske Budejovice	man	62	29.3	Exhibition manager	<LOD	<LOD	High school
16	Ceske Budejovice	woman	61	25.0	Owner	<LOQ	<LOD	High school
17	Ceske Budejovice	woman	41	31.1	Parental leave	<LOD	<LOD	University
18	Ceske Budejovice	man	25	27.7	Medic/Sanitarian	<LOQ	<LOD	High school
19	Ceske Budejovice	man	18	18.6	Guide	<LOD	<LOD	High school
20	Ceske Budejovice	woman	43	26.1	Teacher	<LOD	<LOD	University
21	Ceske Budejovice	woman	40	25.2	Parental leave	128	<LOD	University
22	Ceske Budejovice	woman	59	24.6	Teacher	123	<LOQ	High school
23	Ceske Budejovice	man	19	21.6	Barman	<LOD	<LOD	High school
24	Ceske Budejovice	man	23	23.1	Driver	<LOD	<LOD	Vocationally trained
25	Ceske Budejovice	woman	43	38.1	Social worker	212	<LOD	High school
26	Ceske Budejovice	man	22	22.2	Lifeguard, Rescue worker	<LOD	<LOD	High school
27	Ceske Budejovice	woman	28	21.6	Assistant judge	<LOD	<LOQ	University
28	Ceske Budejovice	woman	24	20.9	Trainee	<LOD	<LOD	University

Sample number	Location	Gender	Age (years)	BMI	Profession	SCCPs (ng/g lw)	MCCPs (ng/g lw)	Education level
29	Ceske Budejovice	man	56	29.4	Production master	<LOD	<LOD	High school
30	Ceske Budejovice	woman	40	21.9	Assistant	<LOD	<LOD	High school
31	Ceske Budejovice	man	43	26.1	Production master	<LOD	<LOD	High school
32	Ceske Budejovice	woman	46	20.4	Sales assistant	<LOD	<LOD	Vocationally trained
33	Ceske Budejovice	woman	59	19.6	Social worker	<LOD	<LOD	High school
34	Ceske Budejovice	man	56	21.3	Teacher	<LOD	341	University
35	Ostrava	man	44	23.3	Worker	337	506	University
36	Ostrava	woman	52	20.5	Managing director	311	352	University
37	Ostrava	woman	42	25.1	Policewoman	653	1064	High school
38	Ostrava	man	31	20.3	Foundry worker in an ironworks	494	486	High school
39	Ostrava	man	30	27.2	Technician	120	<LOQ	Ph.D
40	Ostrava	man	40	24.1	Firefighter	<LOD	<LOQ	High school
41	Ostrava	woman	52	25.1	Pedicurist	<LOD	259	Vocationally trained
42	Ostrava	woman	42	23.3	Teacher	<LOQ	408	University
43	Ostrava	man	39	27.2	Production master	<LOQ	<LOQ	High school
44	Ostrava	man	42	25.9	Head of the powder coating shop	<LOD	794	High school
45	Ostrava	woman	55	28.7	Teacher	197	<LOQ	University
46	Ostrava	woman	50	21.4	Sales assistant	259	<LOQ	University
47	Ostrava	man	22	30.5	Paramedic	<LOQ	<LOQ	High school
48	Ostrava	man	44	25.9	Teacher	<LOD	<LOQ	University
49	Ostrava	woman	38	24.8	Teacher	<LOQ	1529	University
50	Ostrava	woman	25	24.5	General nurse	<LOD	627	University
51	Ostrava	woman	56	21.0	Teacher	161	<LOD	University
52	Ostrava	woman	27	39.0	Nurse	<LOQ	<LOQ	University
53	Ostrava	man	61	21.9	Economist	<LOQ	<LOD	University
54	Ostrava	woman	41	18.5	Assistant	<LOQ	<LOD	University
55	Ostrava	man	27	21.9	Paramedic	<LOD	<LOD	High school
56	Ostrava	man	27	27.8	Sales officer	<LOQ	<LOQ	University
57	Ostrava	man	36	33.0	Head of economics	<LOD	<LOD	University
58	Ostrava	woman	40	22.1	Teacher	<LOD	<LOD	High school

Sample number	Location	Gender	Age (years)	BMI	Profession	SCCPs (ng/g lw)	MCCPs (ng/g lw)	Education level
59	Ostrava	man	64	23.7	HR manager	<LOQ	<LOQ	University
60	Ostrava	woman	56	24.9	Marketing director	<LOQ	<LOD	University
61	Ostrava	man	32	23.8	Economist	<LOD	<LOD	University
62	Ostrava	man	23	35.8	Production operator	<LOD	<LOD	Primary

SCCPs – LOD: 40 ng/g lw, LOQ: 120 ng/g lw;

MCCPs – LOD: 80 ng/g lw, LOQ: 240 ng/lw.
